# Supplementary material for: Splice-Junction-Based Mapping of Alternative Isoforms in the Human Proteome
Source: Cell Rep. Author manuscript; Available in PMC 2020 Jan 15. (PMC6961840; doi:10.1016/j.celrep.2019.11.026)
Supplement: 3 [file NIHMS1546469-supplement-3.zip › DF2/PXD0010154/Testis-39-Q9ULL0-NLQVIVEPKEEEPNLPLVSEEEK.pdf]

A

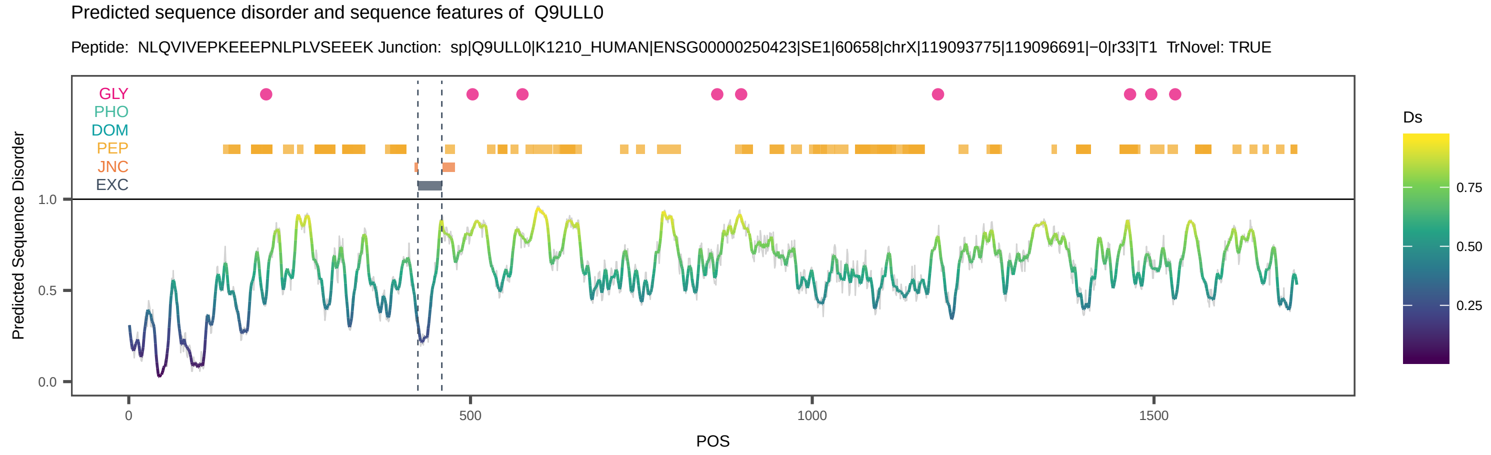

Predicted sequence disorder and sequence features of Q9ULL0
Peptide: NLQVIVEPKEEEPNLPLVSEEEK Junction: sp|Q9ULL0|K1210_HUMAN|ENSG00000250423|SE1|60658|chrX|119093775|119096691|−0|r33|T1 TrNovel: TRUE
GLY
PHO
DOM
PEP
JNC
EXC
Predicted Sequence Disorder
1.0
0.5
0.0
0
500
1000
1500
POS
Ds
0.75
0.50
0.25

B

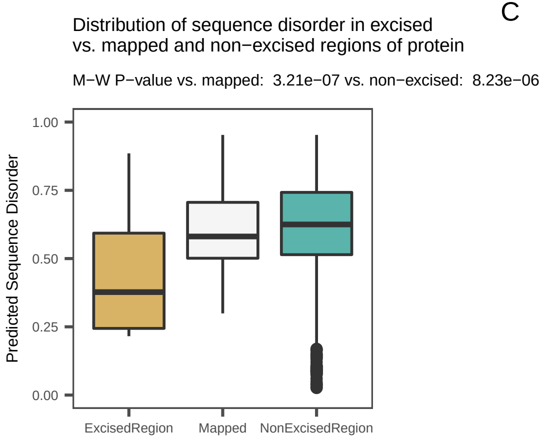

Distribution of sequence disorder in excised
vs. mapped and non−excised regions of protein
M−W P−value vs. mapped: 3.21e−07 vs. non−excised: 8.23e−06
Predicted Sequence Disorder
1.00
0.75
0.50
0.25
0.00
ExcisedRegion
Mapped
NonExcisedRegion

C
